# Ultrasound utilization for implantation of cardiac implantable electronic devices

**DOI:** 10.1007/s00508-023-02215-2

**Published:** 2023-06-23

**Authors:** Muhtashim Mian, Habib Rehman Khan

**Affiliations:** University Hospital, University of Western Ontario, 339 Windermere Rd., N6A 5A5 London, Ontario Canada

**Keywords:** Cardiac resynchronization therapy, Pacemaker, Anesthesia, Lead implantation, Review, Leadless pacemaker

## Abstract

Ultrasound (US) guidance for implantation of cardiac implantable electronic devices (CIED) is currently not routine practice. This article sought to review published data on the use of ultrasound in each of the major surgical steps involved in implantation of CIEDs, including achieving anesthesia, obtaining venous access and implantation of leads. A literature review was performed, revealing a total of 20 peer-reviewed studies that assessed US guidance for CIED implantation; 3 of these were randomized trials while the remainder were mostly feasibility studies. The available data suggest that ultrasound can be useful in guiding implantation of CIEDs, with a trend towards less complication rates; however, more high-quality studies that compare US guidance to traditional techniques in CIED implantation are required.

## Introduction

**Fig. 1:**
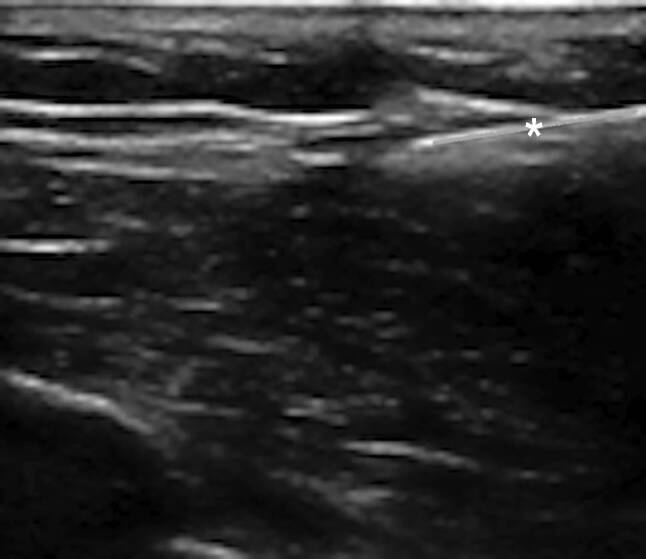
Ultrasound of the soft tissue overlying the chest and the vascular access site. Local anesthetic is injected into the prepectoralis fascia to enable a rapid anesthetic effect (*)

**Fig. 2:**
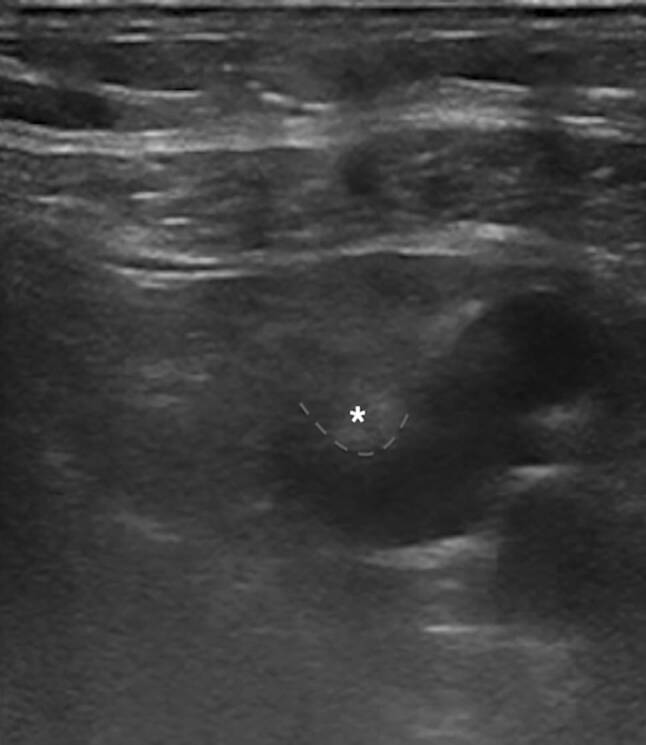
Ultrasound-guided direct access by direct indentation of the axillary vein (*)

**Fig. 3:**
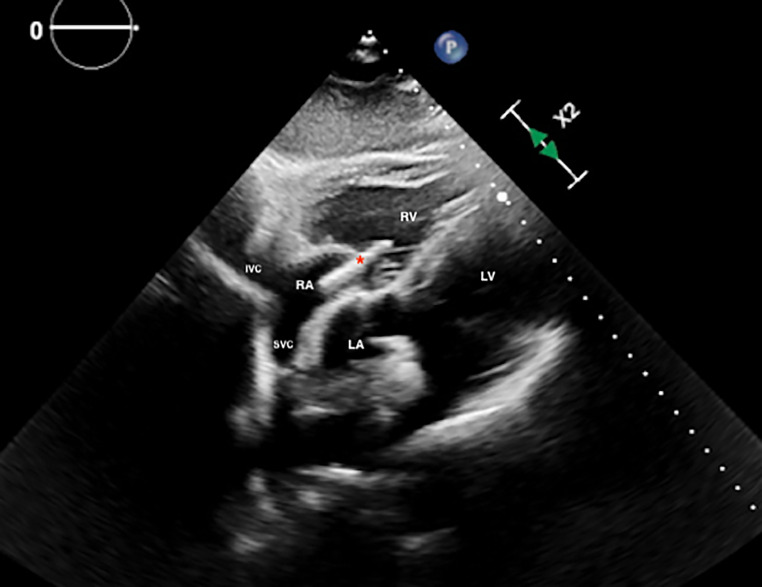
Subcostal view of the heart showing the pacing lead (*) entering through the tricuspid valve into the right ventricle (RV). *IVC* inferior vena cava, *LA* left atrium, *LV* left ventricle, *RA* right atrium, *RV* right ventricle, *SVC* superior vena cava

**Fig. 4:**
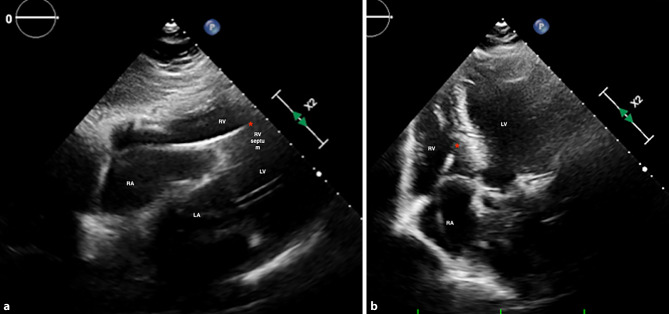
**a** Subcostal view of the heart with the pacing lead (*) opposed to the right ventricular septum (RV septum). **b** Apical 4-chamber view of the heart with the pacing lead (*) deployed to the mid-right ventricle septum. *LA* left atrium, *LV* left ventricle, *RA* right atrium, *RV* right ventricle

Cardiac implantable electronic devices (CIEDs) is a term that encompasses a number of devices that provide treatment for potentially fatal cardiac arrythmia. The CIEDs include devices such as pacemakers, implantable cardioverter defibrillators (ICD), and cardiac resynchronization therapy (CRT). Most CIEDs are implanted under the skin and are connected to the heart via wires (leads) travelling through large veins in the thorax to the heart delivering electrical impulses that regulate the heart’s rhythm. The CIED implantation typically involves active sedation and local anesthesia or rarely general anesthesia, percutaneous cannulation of axillary or a more central vein, guidance of lead(s) through the vein into the right heart chambers with implantation into the endocardium, and finally lead(s) being connected to pulse generator which itself is implanted in a subcutaneous pocket [1]. Figs. 1, 2, 3 and 4 were taken from our group at LHSC and highlight the key steps in implantation of CIEDs using ultrasound.

Pacemakers are implanted transvenously using either single (right ventricle), dual (right atrium and ventricle), or triple lead systems. The CRT is a triple lead pacing technology that improves cardiac function in patients with reduced ejection fraction heart failure with a wide QRS of > 130 ms. In addition to right atrial and right ventricular leads of conventional pacemakers, CRT requires a left ventricle lead that is usually traversed through the coronary sinus with the aid of fluoroscopy. There also exist leadless pacemakers that are implanted directly into the endocardium; their implantation requires the same approach for anesthesia and venous access but subvert the need for leads [2].

The ICDs can be implanted either transvenously or subcutaneously (S-ICDs). S‑ICDs eliminate the need for venous access as the lead is traversed subcutaneously and placed adjacent to the sternum [3]. By virtue of their subcutaneous lead placement, S‑ICDs have the disadvantage of requiring higher energy for defibrillation (with larger generator size) and an inability to provide antitachycardia pacing. An alternative to S‑ICDs was developed called extravascular ICD (EV-ICD) for which the lead was placed substernally to overcome limitations of S‑ICDs [4]. Nevertheless, S‑ICDs and EV-ICDs are mainly defibrillators and cannot provide extended back-up pacing. Theoretically, S‑ICDs and EV-ICDs minimize the risks associated with venous access including endocarditis, pneumothorax and cardiac perforations, although results from meta-analyses have shown mixed results [5–7]. They are often used in patients who have a high risk for complications or in whom vascular assess is not possible [8].

Regardless of transvenous or extravascular approach for implantation of CIEDs, ultrasound (US) can be effectively utilized for most major surgical steps outlined above, including achieving anesthesia in the form of nerve blocks, obtaining venous access, and implantation of lead(s), which are reviewed below. Other implantable devices in cardiology, such as implantable loop recorders which do not provide treatment are not covered in this review.

## Methods

Studies were searched using PubMed and OVID Medline. The search strategy for OVID Medline used a combination of the following terms: cardiac pacing, defibrillator, pacemaker, cardiac resynchronization therapy, leadless, ultrasound. The search yielded 235 articles which were then screened for original research and for relevance to the topic of this review article, namely utilization of ultrasound for anesthesia, vein cannulation or optimal lead placement of CIEDs. The resulting 20 publications were included in this review article.

Figures 1, 2, 3 and 4 were captured during implantation of CIEDs in patients at the London Health Science Center (LHSC). Informed consent was obtained to utilize the ultrasound images for demonstration purposes.

## US-guided anesthesia administration

Most CIEDs are currently implanted using procedural sedation with subcutaneous injection and infiltration of local anesthetic agents. This typically requires patients to fast to minimize intraprocedural aspiration risk. Furthermore, achieving analgesia can be challenging, requiring escalating doses of local anesthetic agents and sedatives which can impair wound healing and negatively impact hemodynamics [9].

Alternatively, US-guided nerve blocks can be used to achieve anesthesia. For instance, pectoral nerve blocks (PECS) involve using US guidance to administer local anesthetic agents in the fascia between the pectoralis major and minor muscles. Outside of CIED implantation, the perioperative use of US-guided nerve blocks in chest wall surgery has demonstrated a reduction in doses of local anesthetics and periprocedural narcotics [10–12].

There are some case reports and feasibility studies assessing US-guided nerve blocks in CIEDs. Antiperovitch et al. demonstrated that a cardioverter-defibrillator could be implanted with only US-guided pectoral and supraclavicular nerve blocks; the patient did not require any sedation or anesthetic, and was pain-free before and after the procedure [13]. A feasibility study by Boyzel et al. showed that only 4 of 36 patients required postprocedural analgesia following implantation of CIED with US-guided PECS nerve block [14]. Similarly, in a non-randomized trial of patients undergoing S‑ICD, Uran et al. showed that combined US-guided serratus anterior plane block and parasternal block required lower doses of local anesthetics and IV analgesia, and less procedural time compared with parasternal block alone or conventional local anesthesia and sedation [15]. In addition to the observed benefits of US-guided nerve blocks in these studies, theoretical benefits include less harmful analgesia to patients who have documented analgesia intolerance, are hemodynamically unstable, older, or those with neuromuscular diseases [16, 17]. Thus, US-guided nerve blocks for implantation of CIEDs is promising but requires more comparative trials to determine noninferiority, if not superiority.

## US-guided venous access

Historically, blind (non-US-guided) subclavian vein cannulation was used to implant CIEDs as it was fast, easy to learn and offered high success rates [18, 19]; however, this approach is associated with higher rates of early (pneumothorax, hemothorax) and late (lead crush) complications [20]. To minimize complications, alternative approaches for vein cannulation were developed including a) fluoroscopy, whereby the axillary or a more proximal vein is cannulated using X‑rays with or without IV contrast for landmarking or b) cephalic vein cut down, whereby the cephalic vein is exposed by surgical dissection of the deltopectoral groove and then cannulated. The use of fluoroscopy has demonstrated higher success rates (~95% vs. 60–85%) and shorter procedure duration time [21]. Cephalic vein cut down reduces the risk of complications compared to subclavian vein cannulation, but no difference was found compared to axillary vein cannulation in meta-analyses [21–23].

US-guided venous access is common practice and is strongly recommended by the American Society of Anesthesiologists particularly for cannulation of internal jugular veins given superior outcomes [24]. At the time of this review, a number of retrospective and prospective (including two randomized controlled trials, RCT) have assessed US-guided axillary vein cannulation (AVC) for implantation of CIEDs (Table 1). Most of these studies showed noninferiority in success rates when comparing US-guided cannulation to traditional forms of cannulation with or without fluoroscopy. Of the two RCTs published, Tagliari et al. showed superiority of US guidance in achieving cannulation, although the comparison was with cephalic vein cut down, which is known to have inferior success rates; interestingly, the complications rates were similar despite cephalic vein cut down being considered the safest form of cannulation [25]. The other RCT published by Liccardo et al. compared US-guided to blind cannulation; this study showed noninferiority in success rates. The remaining comparative studies showed a nonsignificant trend towards less complication rates following US-guided cannulation.

**Table 1:** Studies assessing US-guided vein cannulation for implantation of CIEDs

First author, Year (Ref.)	n	Outcome
**Randomized trial**		
Tagliari et al., 2020 [25]	88	*US-guided AVC (n* *=* *44) vs. cephalic vein dissection (n* *=* *44)* Success = 97.7% vs. 54.5% * Pneumothorax = 2.3% vs. 2.3%
Liccardo et al., 2018 [26]	174	*US-guided AVC (n* *=* *116) vs. blind SCVC (n* *=* *58)* Success = 91.4% vs. 94.8% Pneumothorax = 0% vs. 0% Lead fracture = 1.7% vs. 3.4%
**Retrospective cohort**		
Chandler et al., 2020 [27]	561	*US-guided AVC (n* *=* *187) vs. fluoroscopy-guided AVC/SCVC (n* *=* *374)* Success = 95% vs. 100% Pneumothorax = 0% vs. 1%
Migliore et al., 2020 [28]	95	*US-guided AVC (n* *=* *49) vs. fluoroscopy-guided AVC (n* *=* *46)* Success = 92% vs. 91% Complications = 0% vs. 0%
Lin et al., 2017 [29]	816	*US-guidance with fluoroscopy for AVC (n* *=* *137)* *vs. fluoroscopy-guided/cephalic access (n* *=* *679)* Fluoroscopy time inversely correlated with US use‡ DVT or pneumothorax or hematoma = 2.2% vs. 3.8%
**Feasibility study**		
Deluca et al., 2020 [30] (retrospective)	548	*US-guided AVC* Success = 99.8% Pneumothorax = 0.5% Lead crush = 0%
Ahmed et al., 2020 [31] (retrospective)	166	*US-guided AVC* Success = 93% Pneumothorax = 0% Hematoma = 0.01%
Clark et al., 2018 [32] (retrospective)	16	*US-guided AVC* Success = 89% Immediate complications = 0%
Esmaiel et al., 2016 [33] (retrospective + prospective)	403	*US-guided AVC* Success = 99.3% Hematoma = 0.5% Other complications = 0%
Nash et al., 1998 [34] (Prospective)	70	*US-guided SCVC* Success = 80% Complications = 0%

*AVC* axillary vein cannulation, *SCVC* subclavian vein cannulation, *US* ultrasound

*p < 0.05

‡ Significant inverse correlation (*p* < 0.001) that became non-significant when comparison adjusted for age and number of leads placed

Although US-guided cannulation for CIED implantation is becoming more commonplace, it is not yet practiced in the majority of centers or guideline-based. While the studies outlined in this review lend support for US-guided axillary cannulation for venous access in CIEDs, more studies, particularly randomized controlled trials are needed.

## US-guided lead implantation, assessment and extraction

Although the number of published studies are few, transthoracic echocardiography (TTE) has been used for the placement of temporary transvenous pacing leads in various clinical settings (Table 2). To our knowledge, no large studies have thus far evaluated real-time US-guided placement of leads for permanent pacemakers. A case report of a full implant by TTE was reported recently with a good example of how the US can aid lead guidance past the tricuspid valve into the right ventricular septum [35]. A recently published case series of a single operator experience demonstrated safe use of ultrasound for the entire duration of single chamber CIED implants in a selected population [36]. However, Saba et al. demonstrated that speckle tracking echocardiography could be used to identify sites of last activation of myocardium and thus guide optimal implantation of left ventricle (LV) lead using fluoroscopy for CRT; in their 2013 RCT (*n* = 187), patients enrolled in the echocardiography-guided LV lead placement group had a significantly longer time to heart failure hospitalization or death compared to the routine fluoroscopy group (hazard ratio, HR 0.48, confidence interval, 95% CI 0.28–0.82, *p* = 0.006) [37].

**Table 2:** Studies assessing utilization of TTE for implantation of temporary pacing leads

First author (Ref.)	Study type	n	Outcome
Ferri et al., 2016 [38]	Prospective cohort (ER setting)	203	*TTE-guided temporary lead implantation (n* *=* *113)* *vs. fluoroscopy-guided temporary lead implantation (n* *=* *90)* Success = 100% vs. 100% Procedure time (s) = 291 (CI 241–423) vs. 343 (CI 213–465) Time to pacing (min) = 22 (CI 19–39) vs. 43 (CI 23–55)* Electrode malfunction/dislodgment = 8.8% vs. 8.9% Device-related infections = 2.7% vs. 11.1%*
Pinneri et al., 2013 [39]	Prospective cohort (ICU, CCU, ER setting)	106	*TTE-guided temporary lead implantation (n* *=* *53)* *vs. fluoroscopy-guided temporary lead implantation (n* *=* *53)* Success = 100% vs. 100% Time to pacing = 439 ± 179 s* vs. 716 ± 235 s Electrode malfunction/dislodgment = 5.7%* vs. 22.8%
Sajuas et al., 2019 [40]	Retrospective feasibility (perioperative)	36	*TTE-guided placement of temporary transvenous catheter* Success = 97.2% Complications = 0%
Aguilera et al., 2000 [41]	Prospective feasibility (ER setting)	8	*TTE-guided placement of temporary transvenous catheter* Success = 88.9% Complications = 0%
Jesus et al., 1992 [42]	Prospective feasibility (CCU setting)	20	*TTE-guided temporary lead implantation* Success = 95% Lead dislodgement = 5% Complications = 0%

*TTE* transthoracic echocardiography, *ER* Emergency Room, *CCU* Coronary Care Unit, *ICU* Intensive Care Unit, *CI* Confidence Interval

*p < 0.05

Separately, lead assessment and extraction has been demonstrated with ultrasound guidance. Beaser et al. showed that using TTE among 60 patients, the degree of ultrasound-graded intravascular lead adherence correlated significantly with difficulty of lead extraction [43]. Transesophageal echocardiography (TEE) can also be used; during lead extraction of CIEDs, TEE can identify potential complications as well as guide management if complications arise [44, 45].

## Conclusion

The use of ultrasound is a relatively recent technique to improve the deliverance of anesthesia in the form of nerve blocks, for cannulation of axillary vein, and for implantation of temporary CEID leads. The data suggest great potential for using ultrasound in CEID implantation and management. US-guidance might become more necessary as pacemaker techniques require more precision, for instance, in conductive system pacing where good visualization of the interventricular septum is beneficial during lead implantation. The dearth of literature in this field is likely multifactorial, including the difficulty in trial design (blinding, maintaining clinical equipoise) and perhaps the perception that fluoroscopy guidance is reliable and good enough; however, use of fluoroscopy can result in high expenses associated with using fluoroscopy machines and protecting medical professionals from potential musculoskeletal injuries due to extended periods of wearing radiation protection gear. More studies are actively recruiting to evaluate the feasibility of US guidance CEID implantation such as the RADICAL USE study (NCT04858698). Further studies should be undertaken to discern the comparative effectiveness of US-guided versus traditional CEID implantation alongside surgeon training to get familiar with US views that will assist in the procedures.
